# Magnesium isotope geochemistry of the carbonate-silicate system in subduction zones

**DOI:** 10.1093/nsr/nwac036

**Published:** 2022-02-26

**Authors:** Shui-Jiong Wang, Shu-Guang Li

**Affiliations:** State Key Laboratory of Geological Processes and Mineral Resources, and Institute of Earth Sciences, China University of Geosciences (Beijing), Beijing 100083, China; State Key Laboratory of Geological Processes and Mineral Resources, and Institute of Earth Sciences, China University of Geosciences (Beijing), Beijing 100083, China; CAS Key Laboratory of Crust-Mantle Materials and Environments, School of Earth and Space Sciences, University of Science and Technology of China, Hefei 230026, China

**Keywords:** magnesium isotope, fractionation, deep carbon cycle, subduction, carbonate

## Abstract

The lighter magnesium (Mg) isotopic signatures observed in intraplate basalts are commonly thought to result from deep carbonate recycling, provided that the sharp difference in Mg isotopic composition between surface carbonates and the normal mantle is preserved during plate subduction. However, deep subduction of carbonates and silicates could potentially fractionate Mg isotopes and change their chemical compositions. Subducting silicate rocks that experience metamorphic dehydration lose a small amount of Mg, and preserve the original Mg isotopic signature of their protoliths. When the dehydrated fluids dissolve carbonate minerals, they may evolve into lighter Mg isotopic compositions. The solubility of carbonate minerals in fluids decreases in the order of calcite, aragonite, dolomite, magnesite and siderite, leading to selective and partial dissolution of carbonate minerals along the subduction path. At the island arc depth (70–120 km), the metamorphic fluid dissolves mainly Mg-poor calcites, and thus the fluid has difficulty modifying the Mg isotopic system of the mantle wedge and associated arc basalts. At the greater depth of the back arc system or continental margin (>150 km), the supercritical fluid can dissolve Mg-rich carbonate minerals, and its interaction with the mantle wedge could significantly imprint the light Mg isotopic signature onto the mantle rocks and derivatives. Meanwhile, the carbonate and silicate remaining within the subducting slab could experience elemental and isotopic exchange, during which the silicate can obtain a light Mg isotopic signature and high CaO/Al_2_O_3_, whereas the carbonates, particularly the Ca-rich limestone, shift Mg isotopes and MgO contents towards higher values. If this isotopic and elemental exchange event occurs widely during crustal subduction, subducted Ca-rich carbonates can partially transform into being Mg-rich, and a portion of recycled silicates (e.g. carbonated eclogites) can have light Mg isotopic composition alongside carbonates. Both serve as the low-δ^26^Mg endmember recycled back into the deep mantle, but the latter is not related to deep carbonate recycling. Therefore, it is important to determine whether the light Mg isotopic signatures observed in intraplate basalts are linked to deep carbonate recycling, or alternatively, recycling of carbonated eclogites.

## Teaser

This paper reviews Mg isotope geochemistry of silicate and carbonate during plate subduction. Ca-Mg exchange between carbonates and silicates, and selective partial dissolution of carbonates during crustal subduction are to be considered when applying Mg isotopes to trace deep carbon cycle.

## INTRODUCTION

A magnesium (Mg) isotopic system has been applied to trace the deep recycling of carbonates [1] for three broad reasons. First, surface carbonates, regardless of inorganic or organic origin, have remarkably lighter Mg isotopic compositions than terrestrial silicates [2,3]. This suggests that an injection of carbonates into the mantle has the ability to cause mantle Mg isotopic heterogeneity. Second, igneous processes such as mantle melting, degassing and crystallization produce negligible Mg isotope fractionations [4–6], such that the Mg isotopic signature of mantle sources can be directly seen from their derivative basalts. Finally, crustal subduction seems not to erase the contrasting Mg isotopic signature between sedimentary carbonates and silicates [7]. While the last statement is empirically accepted [1], the behavior of Mg isotopes in a subduction zone is complicated and relatively less well constrained. Attempts have been made over the last decade to decipher the magnitude and mechanism of Mg isotope fractionation by subduction-related processes [7,8]. It helps to answer some fundamental questions, for example: (i) is carbonate the only low-δ^26^Mg carrier among those recycled into the mantle? (ii) Can the composition and solubility of carbonate be changed during subduction? (iii) Why can the low-δ^26^Mg signature be observed in intraplate basalts but not in island arc basalts?

This contribution, built upon materials presented in previous reviews and incorporating the findings of the most recent studies [9–11], aims to provide an overview of the behavior of carbonate-silicate systems and their Mg isotopes in subduction zones. We examine the physical and chemical properties of the subducting silicate-carbonate package during crustal subduction in the first section of this article. In the second section, we evaluate how subduction-related processes could affect the Mg isotopic system and the chemical composition of subducting carbonate and silicate. In the third section, we put these fractionation events in the context of a plate tectonic framework to explore the robustness of linking Mg isotopic anomalies in mantle-derived rocks to carbonate recycling.

## CARBONATE-SILICATE PACKAGE IN SUBDUCTION ZONE

**Figure 1.**
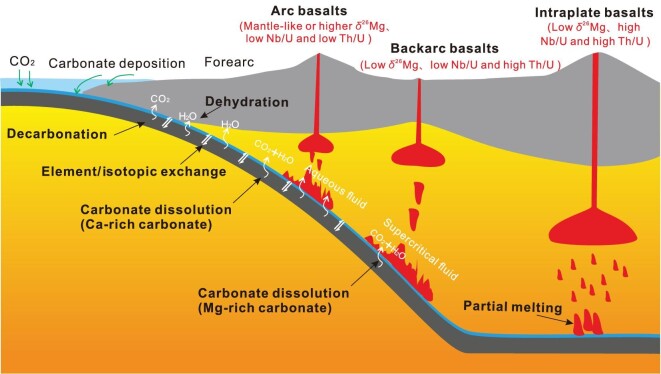
Cartoons showing some of the important processes related to the subduction of the silicate-carbonate package into the deep mantle (not to scale). Please see the main text for details.

The carbonates that enter into the trench are mainly from the platform carbonates on the overriding plate and marine carbonates precipitated on the oceanic floor [12]. They are carried by the subducting plate, together with the silicates, to the deep mantle. The subducting carbonate-silicate package experiences significant changes in physical and chemical properties, leading to carbon mobility and potential isotope fractionations. Processes of particular interest are summarized below (Fig. 1).

### Metamorphic dehydration

At elevated pressures and temperatures in subduction zones, the fluid in the pore spaces of rocks, boundaries between crystals, or in hydrous minerals, will be liberated due to compression and metamorphism. With increasing metamorphic grade, for example, from sub-greenschist- to greenschist-, amphibolite- and eclogite-facies, the amount and chemical composition of the dehydrated fluid vary as a function of the lithologies and dehydration reactions [13]. The dehydrated fluid then migrates upwards due to its low density and viscosity compared to the surrounding rocks. The importance of metamorphic dehydration is 2-fold. First, metamorphic dehydration may cause loss of Mg along with phase changes. If subducting rocks, after metamorphic dehydration, display different Mg isotopic compositions from their protolith, the extent and magnitude of such isotope fractionation must be calibrated. Second, the fluids may change from aqueous fluid to supercritical fluid as pressure and temperature increase during crustal subduction, and they play a key role in mass transfer and elemental/isotopic exchanges in subduction zones [14,15]. Extensive fluid–rock interactions in subduction zones could facilitate carbon mobility through a series of reactions such as decarbonation, carbonate dissolution and carbonate-silicate reaction [16–18]. We are interested in whether the fluid has a similar Mg isotopic composition as its protolith, whether Mg isotopes fractionate during carbon-mobility events and whether the chemical composition and solubility of the subducting carbonate change during plate subduction.

### Decarbonation

Metamorphism of carbonate rocks may cause decarbonation via the reactions of CaCO_3_ + SiO_2_ = CaSiO_3_ + CO_2_, or CaMg(CO_3_)_2_ + 2SiO_2_ = CaMg(SiO_3_)_2 _+ 2CO_2_. Given the strong dependence of decarbonation on pressure, temperature and composition, the degree of decarbonation changes among subducted lithologies, varies from one subduction to another, and may differ significantly between Precambrian and modern subductions. Based on phase equilibria computation of the metamorphic decarbonation of subducting slabs, Kerrick and Connolly [19] proposed that along typical subduction geotherms metamorphic decarbonation is unlikely to happen and its effect on transferring CO_2_ from subducting slabs to arc magmas is negligible. In general, decarbonation is more efficient in carbonated sediments relative to carbonated basaltic rocks and siliceous limestones [19,20]. In rare cases where subduction geotherms are high, for example the subduction of young oceanic crust at slow convergent rates, decarbonation is feasible in the forearc regions (because of low pressure and relatively high temperatures) [20]. This raises the possibility that decarbonation may have been high or complete at Precambrian subduction zones at which the temperature was as much as ∼100°C higher than the hottest present-day subduction zone [21]. The infiltration of H_2_O-rich fluid could also promote decarbonation of subducted marine sediments [19,22]. Stewart and Ague [18] predict that 40%–65% of the CO_2_ in subducting crust can be released via metamorphic decarbonation at forearc depths, which is remarkably higher than previously thought. In addition, natural observations and experimental studies found that carbonate minerals can be reduced to form hydrocarbons in subduction-zone settings under low oxygen fugacity, with the residual mineral assemblage consisting of iron-bearing dolomite, magnetite and graphite [23]. According to the decarbonation reactions, Mg isotopic signatures of carbonates would be inherited by newly formed silicates during decarbonation.

### Carbonate dissolution

Metamorphic fluid is probably the most important agent mobilizing carbon from the subducting slab to the arc mantle [17]. Fluid inclusions in subduction-related ultrahigh metamorphic rocks contain a range of carbonate minerals, suggesting that a substantial amount of carbonate minerals can be dissolved in metamorphic fluids [17,24,25]. Ague and Nicolescu [17] investigated the alteration of the exhumed Eocene Cycladic subduction complex on the Syros and Tinos islands, Greece, and found that the abundance of Ca-rich carbonate decreases drastically from the marble to the fluid conduits, suggesting that up to 60%–90% of the CO_2_ was released from the rocks by fluid. Theorical and experimental studies now find that metamorphic fluids in subduction zones may transport significant quantities of carbonate minerals, with the solubility of carbonate minerals in the order of calcite > aragonite > dolomite > magnesite > siderite [16,26,27]. As a result, aqueous fluids selectively dissolve Ca-rich carbonate at the forearc and arc mantle depth (70–120 km) leaving Mg-rich carbonates in the subducting slabs [1]. At back arc or continental margin depths (≧150 km), supercritical fluids derived from subducting slabs are capable of dissolving Mg-calcite and dolomite [24,25]. The solubility of carbonate minerals increases as the subducting slab goes deeper and the salinity of fluid composition increases. For example, Shen *et al*. [25] found abundant carbonate mineral inclusions including calcite, dolomite and magnesite, in metamorphic zircons precipitated from supercritical fluids. Given the distinct Mg isotopic signature of Ca-rich and Mg-rich carbonates [11], selective and partial dissolution of subducted Ca-rich carbonates can potentially lead to different Mg isotopic compositions of the evolved fluids.

### Partial melting

Another important process that could mobilize carbon in subduction zones is the melting of carbonate-bearing rocks. The fate of subducted carbonates that survived decarbonation and dissolution at forearc and arc depth hinges on the location of the solidus of carbonated rocks relative to the thermal structure of the subduction zone. Experimental studies have determined the solidi of three dominant carbonated lithologies: carbonated ocean floor sediments, carbonated altered basalt and carbonated peridotite [28–35]. Carbonated sediments have the lowest solidi and thus are more prone to losing carbon during subduction. Grassi and Schmidt [28] suggested that carbonated sediments may melt at two depths of the subducting slabs: 6–9 GPa and 20–22 GPa. At any given pressure, the solidi of carbonated oceanic floor basalts and carbonated peridotites are on average higher than that of carbonated sediments, and remain hotter than the slab-top condition of most modern subduction zones [36]. Pure carbonate rocks have an even higher melting temperature than carbonated silicates [37]. Therefore, carbonated oceanic floor basalts and carbonates are the two major carbon carriers in the subducting slabs. Isotopic studies suggest that the carbon in the subducting slabs could be introduced to the mantle transition zone (410–660 km) [1]. Recently, Thomson *et al*. [33] determined the melting phase relations of a synthetic mid-ocean ridge basalt (MORB) composition containing 2.5 wt% CO_2_ between 3 and 21 GPa, and found that the melting curve of carbonated oceanic crust will intersect the majority of slab geotherms at depths of 300–700 km, leading to the idea that melting at this depth would create a barrier to direct carbonate recycling into the lower mantle. Melting of recycled carbonated rocks could contribute to the formation of intraplate basalts, which in turn could impart their distinct Mg isotopic signature to the mantle melts.

### Carbonate-silicate reaction

As previously discussed, thermodynamic modeling of the devolatilization of carbonate-bearing subducting slab and melting experiments point towards the preservation of solid carbonates along geotherms of modern subduction zones [38]. The carbonate minerals interact chemically with silicates during subduction and undergo changes in both physical and chemical properties. Kushiro [39] studied carbonate-silicate interaction at pressures between 2.3 and 7.7 GPa and temperatures between 800 and 1400°C. They found that calcite is unstable in the presence of enstatite, and reacts with enstatite to form dolomite and diopside. Studies predict that the carbonate mineral stable at shallow depth is calcite-rich, at intermediate depth is dolomite-rich and at greater depth is magnesite-rich [36], suggesting that carbonate carried by a subducted plate mainly resides in MgCO_3_ throughout much of the mantle via forward reaction CaCO_3_ + MgSiO_3_ = MgCO_3_ + CaSiO_3_. Therefore, the above silicate-carbonate interaction could transform calcite to magnesite so that it fixes carbon in the subducting slabs in the form of more stable Mg-rich carbonate minerals. The carbonate-silicate interaction during crustal subduction may induce massive elemental and isotopic exchange.

## MAGNESIUM ISOTOPE FRACTIONATION DURING SUBDUCTION

The preceding discussion introduces the subduction-related processes that could potentially mobilize carbon and fractionate Mg isotopes. In this section, we review recent advances with regard to the behavior of Mg isotopes in these processes.

### Mg isotopic compositions of metamorphic rocks

Mg preferentially partitions into a solid during metamorphic dehydration, leading to lower Mg concentrations of the fluid relative to the source. Typical subduction-zone fluids have Mg concentrations (0 to 125 mmol/kg) lower than seawater (average 50 mmol/kg) [13]. This is consistent with the results of many experimental studies on elemental partitioning between fluid and minerals during dehydration of sedimentary and basaltic rocks [40–42], which yield distribution coefficients of Mg (D_solid/fluid_) in the range of 0.7 to 70 [40–42]. Assuming that a rock contains 5% of fluid that is sequentially lost during dehydration, the dehydrated fluid takes away only <7% of the bulk-rock Mg. Isotope fractionation through metamorphic dehydration (ϵ_fluid-solid_) has not been experimentally determined yet. However, mass balance calculation suggests that a variation of ϵ_fluid-solid_ from −1.0 ‰ to +1.0 ‰ would result in a dehydration-induced shift of δ^26^Mg in solid smaller than 0.04 ‰, within current analytical uncertainties for δ^26^Mg.

The inferred lack of Mg isotope fractionation in rocks during metamorphic dehydration is supported by Mg isotopic analyses on metamorphic rocks. Wang *et al*. [43] measured Mg isotopic compositions of a suite of metabasalts from the Dabie-Sulu orogen, Eastern China, all of which share the same protolith. These samples include greenschist, amphibolite and eclogites, representing products of prograde metamorphism in the subduction zone. Despite the decreasing loss of ignition (LOI) with increasing metamorphic grade, the metabasalts have similar Mg isotopic compositions. Therefore, metamorphic dehydration has a limited effect on Mg isotopic systematics in metabasalts. A similar conclusion has been reached by Li *et al*. [44] and Teng *et al*. [45] who found that high-grade metamorphic granulites and eclogites have homogeneous mantle-like δ^26^Mg values as their protoliths. Metamorphic dehydration of sedimentary rocks causes limited Mg isotopic changes in bulk rock as well. Metapelites exposed in the Irvea zone, Italy, represent a typical prograde metamorphic sequence from middle amphibolite- to granulite-facies. The Mg isotopic compositions of these metapelites do not vary with metamorphic grade but are inherited from the source heterogeneity [46]. Li *et al*. [47] studied metapelites from the Onawa contact aureole, Maine. They documented that metapelites across the aureole, with increasing metamorphic grade, from the outmost chlorite zone to the andalusite-cordierite zone, potassium feldspar zone, sillimanite zone and leucocratic-vein zone, have similar Mg isotopic compositions. Both studies conclude that prograde metamorphic dehydration causes limited Mg isotopic changes in metapelites. Additionally, Wang *et al*. [48] studied the behavior of Mg isotopes at even lower-grade metamorphic conditions where devolatilization might be larger. The mudrocks studied by Wang *et al*. [48] experienced diagenesis to sub-greenschist metamorphism. The generally heavy Mg isotopic compositions of the mudrocks are not related to metamorphic dehydration but are inherited from their sources [48].

**Figure 2.**
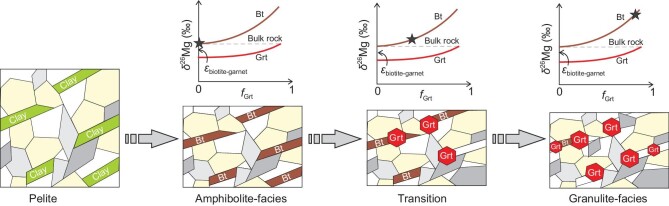
Cartoon showing the redistribution of Mg isotopes during prograde metamorphism of metapelites from the Irvea zone. ϵ_biotite-garnet_ represents the Mg isotope fractionation between biotite and garnet. At amphibolite-facies, the biotite (Bt) is the dominant Mg host of the metapelites, and its δ^26^Mg value is close to the bulk-rock value. At granulite-facies, the garnet (Grt) becomes the dominant Mg host of the metapelites, and its δ^26^Mg value approaches the bulk-rock value with biotite having δ^26^Mg offset from the garnet by ϵ_biotite-garnet._

Despite a limited shift in Mg isotopic composition at the bulk-rock scale during prograde metamorphic dehydration, massive redistribution of Mg occurs among metamorphic minerals accompanied by large Mg isotope fractionations. One typical example comes from the metapelites in the Irvea zone [46]. Biotite and garnet are the two major Mg hosts in these metapelites. During the prograde metamorphic reaction of biotite + sillimanite + plagioclase + quartz → garnet + K-feldspar + rutile + melt, the mineralogy of metapelites changes from being biotite dominated at amphibolite-facies to garnet dominated at granulite-facies (Fig. 2). Due to large inter-mineral Mg isotope fractionation between biotite and garnet (Δ^26^Mg_Bt-Grt_ = 0.96 × 10^6^/T^2^), the mineral δ^26^Mg values, as expected from mass balance, increase with increasing metamorphic prograde (Fig. 2).

Overall, the Mg isotopic compositions of subducted silicate rocks preserve their protolith's signature. Subducted carbonates after metamorphism may experience decarbonation, dissolution and isotopic exchange, whose effects on the Mg isotopic system will be discussed below.

### Mg isotopic compositions of dehydrated fluids

Dehydrated fluids from silicate rocks have highly variable and generally heavier Mg isotopic compositions relative to their source rocks under high pressure-temperature (P-T) metamorphic conditions. The fluid-precipitated high pressure (HP) quartz veins in the Dabie orogen represent the fluids derived from dehydration of the metabasalt. They have higher δ^26^Mg values (from +0.08 to +0.15‰) relative to their basaltic protolith (−0.25±0.04‰) [49]. The coesite-bearing white schist at Dora-Maira in the Western Alps is characterized by strong Mg enrichment, which could be caused by infiltration of Mg-rich fluid derived from dehydration of serpentinites. Chen *et al*. [50] found that these white schists (T = 730°C and 4.0 GPa) have extremely heavy Mg isotopic compositions (δ^26^Mg up to +0.72‰), and suspected that the fluid could be derived from the breakdown of Mg-rich hydrous minerals such as talc and antigorite in serpentinite at the slab–mantle interface. The heavy Mg isotopic compositions (with δ^26^Mg up to +0.61‰) of the coesite-bearing jadeite quartzites from the Dabie orogen are also interpreted as being a result of the infiltration of fluid dehydrated from the breakdown of biotite in subducted metasedimentary rocks [51]. The retrograde eclogites and blueschists from southwestern Tianshan have interacted with metamorphic fluids mainly derived from subducting sediments in the subduction channel [52]. Geochemical proxies of the eclogites and blueschists allow us to distinguish two components of the fluid. One is high-large-ion lithosphile elements (LILEs) fluid derived from dehydration of mica-group minerals. The other has higher Pb concentration and ^87^Sr/^86^Sr relative to typical oceanic basalts, suggesting that the fluid is likely released from dehydration of epidote-group minerals. The fluid derived from mica dehydration contains a considerable amount of Mg that is isotopically heavy [46], and thus shifted the δ^26^Mg of retrograde eclogites towards higher values. The fluid derived from epidote dehydration has little Mg so as not to influence the Mg isotopic system of the retrograde eclogites.

Given the similar octahedral coordination environment of Mg-O in between common metamorphic hydrous minerals (e.g. biotite and hornblende) and fluid [Mg(H_2_O)_6_)]^2+^, the isotope fractionation by dehydration of hydrous minerals might be small. It is suggested that the Mg isotopic systematics of these fluids are mainly determined by the hydrous minerals from which they derive. If the hydrous minerals control the Mg isotopic composition of the dehydrated fluids, a further question arises: why do the hydrous minerals in metamorphic rocks have heterogeneous and generally heavier Mg isotopic compositions? First, most hydrous minerals in metamorphic rocks were transferred from clay minerals that are products of surface water–rock interaction. Surface chemical weathering produces large Mg isotope fractionations, leading to the incorporation of heavy Mg isotopes into clays in the weathering residue [53]. Second, as inferred by the Irvea zone metapelites, it is highly likely that the hydrous mineral's δ^26^Mg value increases with increasing metamorphic grade (Fig. 2), given the massive redistribution of Mg among metamorphic minerals and potentially large inter-mineral isotope fractionation between hydrous minerals and newly formed metamorphic minerals such as garnet. Take the biotite in metapelites from the Ivrea zone as an example: the δ^26^Mg of biotite increases from −0.08 ‰ at amphibolite-facies to +1.10 ‰ at granulite-facies. As a result, metamorphic fluids derived from biotite dehydration could have highly variable Mg isotopic compositions that are closely correlated to the metamorphic grade (Fig. 2).

As a note, this section only mentions the primary fluid dehydrated from silicates. When the fluid travels and interacts with carbonates, the Mg isotopic composition will change as discussed below.

### Carbonate dissolution on the Mg isotopic systematics of metamorphic fluids

**Figure 3.**
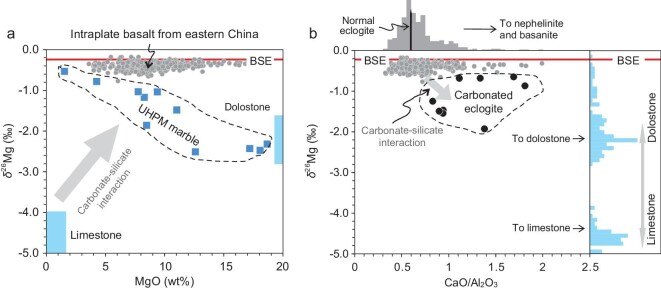
A compilation of the Mg isotopic composition of sedimentary carbonates, intraplate basalts from eastern China, normal and carbonated eclogites, and ultrahigh pressure metamorphic (UHPM) marbles. The UHPM marbles display a negative correlation between δ^26^Mg and MgO after interacting with the concomitant silicate during crustal subduction (a). The carbonated eclogites after interacting with the carbonate during crustal subduction have low δ^26^Mg and high CaO/Al_2_O_3_, which is distinct from that of the normal eclogites (b). The histogram of sedimentary carbonate is modified from Ref. [11]; Mg isotopic data of normal/carbonated eclogites and UHPM marbles are from Ref. [54]; data for intraplate basalts are from Refs [1,55–59]. The Bulk Silicate Earth value is from Ref. [4].

When the metamorphic fluid dissolves carbonate minerals, the Mg isotopic composition of the fluid may become lighter. Sedimentary carbonate rocks range from Ca-rich limestone to Mg-rich dolomite. Ca-rich carbonate minerals have generally lighter Mg isotopic compositions than Mg-rich carbonate minerals (Fig. 3). At forearc and island arc depths, metamorphic aqueous fluids dissolve mainly calcite and, to a lesser extent, dolomite [1,16,17,26]. As temperature and pressure increase at the back arc or continental margin depth (>150 km), the supercritical fluids with high solubilities of trace elements and carbonate minerals are able to dissolve dolomite and magnesite [25]. Given selective dissolution of carbonate minerals during subduction, the fluid may have different Mg isotopic compositions depending on the solute.

Chen *et al*. [60] measured Mg isotopic compositions of the jadeitites from Myanmar. These white jadeitites were precipitated from Na-Al-Si fluids at the forearc slab-mantle interface (1–1.5 GPa, and 300–500°C). They are characterized by extremely light Mg isotopic compositions (δ^26^Mg = −0.55 to −0.92‰) that are negatively correlated with CaO/TiO_2_ and CaO/Al_2_O_3_ ratios. Chen *et al*. [60] proposed that the high-salinity reduced fluid dehydrated from subducting slabs enhanced the dissolution of Ca-rich carbonates that eventually lowered the δ^26^Mg values of fluids. Chen *et al*. [61] studied high-pressure metamorphic leucophyllites from the Eastern Alps. They were formed under similar pressure but higher temperature than the jadeitites in Myanmar (500–600°C), and thus are capable of dissolving Mg-rich calcite at the forearc depth. Two types of fluids are recognized in terms of Mg isotopes. One has high δ^26^Mg values (>0.3‰), which is likely from dehydration of talc-rich serpentinite, and the other has extremely low δ^26^Mg values (<−1.3‰), likely produced by dissolution of mainly Mg-calcite at forearc conditions. Shen *et al*. [25] studied the Maowu ultramafic massif, which represents an exhumed fragment of mantle wedge from the Dabie orogen, with peak metamorphism of 5.3–6.3 GPa and 800°C. The garnet pyroxenite within the Maowu ultramafic massif was formed by mantle metasomatism of supercritical fluids derived from subducting slabs, and is characterized by high Th/U ratios (up to 23) and light Mg isotopic compositions (δ^26^Mg down to −0.99‰). Abundant carbonate mineral inclusions, including calcite, dolomite and magnesite, have been found in metamorphic zircons formed from supercritical fluids. The supercritical fluids have high Mg content and light Mg isotopic compositions as they dissolve a considerable amount of Mg-rich carbonate minerals [25]. When traveling and interacting with the mantle wedge, the supercritical fluids impart light Mg isotopic signatures and high Th/U to the Maowu garnet pyroxenite [25].

### Mg isotope fractionation during decarbonation

Decarbonation during modern subduction may be negligible, but could be facilitated in the presence of fluid [18]. Decarbonation releases CO_2_ while leaving Mg and Ca in the silicate. This reaction could lead to the newly formed silicate enriched in light Mg isotopes and high CaO/Al_2_O_3_. Shen *et al*. [62] analyzed the Mg isotopic composition of endoskarn xenoliths from the Sierra Nevada batholith in California, and found that the pyroxenite rim, which is the product of the decarbonation reaction, is characterized by light Mg isotopic composition and high CaO/Al_2_O_3_. The Mg isotopic anomalies can be explained by the mixing of Mg between granodioritic magma and dolomitic wallrock. Decarbonation of the dolomitic wallrock transfers the Mg isotopic signature from carbonate to silicate. In the Precambrian subduction where the geothermal gradient was higher, decarbonation may have been significant. It is possible that subducted carbonates would have been completely decarbonated leaving light Mg isotopes to the subducting silicates.

### Mg isotopic exchange between carbonate and silicate

The large Mg isotopic difference between surface carbonate and silicate will be reduced at elevated temperatures during crustal subduction, if the equilibrium isotope fractionation rule (Δ^26^Mg = A × 10^6^/T^2^) applies. The experimental study using a three-isotope method found that equilibrium Mg isotope fractionation between magnesite and forsterite follows the equation Δ^26^Mg_forsterite-magnesite_ = 0.06 (±0.04) × 10^6^/T^2^ at high temperatures [63], that is, 0.44 ± 0.10‰ at 600°C. These experimentally determined high-temperature equilibrium fractionation values are significantly smaller than the apparent isotopic difference observed at the surface environment (Fig. 3). Whether or not complete isotopic equilibrium between coexisting carbonate and silicate can be achieved during crustal subduction is uncertain, but massive diffusion-induced isotopic exchange between the two lithologies is expected.

Eclogite boudins enclosed in the ultrahigh metamorphic marbles in the Rongcheng area, Sulu orogenic belt, have chemically interacted with the host marble during high-pressure metamorphism. Wang *et al*. [54] found that the eclogite boudins have extremely low δ^26^Mg and high δ^18^O values, which is in sharp contrast to the normal eclogites in the Sulu orogen. The ultrahigh-pressure metamorphic marbles show negative correlation between δ^26^Mg and MgO/CaO, which is opposite to their protoliths, in which dolostones have heavier Mg isotopic composition than limestones (Fig. 3). These Mg and O isotopic anomalies, observed in both eclogite boudins and marbles, are interpreted as a result of elemental and isotopic exchange during crustal subduction. The big difference in Mg content between limestone and dolostone results in differential Mg isotopic exchange against eclogites boudins. The Mg-poor limestone that suffered extensive elemental and isotopic exchange has its δ^26^Mg and MgO contents elevated significantly, whereas the Mg-rich dolostone retains its original δ^26^Mg values because of its high-Mg nature (Fig. 3a). The eclogitic minerals, after elemental and isotopic exchange, obtain light Mg isotopic and high CaO/Al_2_O_3_ signatures (Fig. 3b). The carbonate-silicate interaction during crustal subduction is of particular consequence. First, the carbonated eclogites, after isotopic exchange, can have low δ^26^Mg values down to −1.93‰ and high CaO/Al_2_O_3_ up to 1.81 (Fig. 3b). Recycling of these components can produce Mg isotopic heterogeneity of the mantle domains but it is not directly related to carbonate recycling. Second, the carbonates, after isotopic exchange, rearrange the δ^26^Mg vs. MgO array (Fig. 3a), and thus the endmember of carbonates recycled into the deep mantle is mainly Mg-rich dolostone and magnesite.

## LINKING THE MG ISOTOPIC SYSTEM TO RECYCLED CARBONATE

The above-mentioned Mg isotopic geochemistry in the subduction zone proves that multiple subduction-related processes can change the Mg isotopic system of subducting silicate and carbonate. Understanding the behavior of Mg isotopes at different stages of crustal subduction can place constraints on the robustness of linking Mg isotopic anomalies in mantle-derived rocks to carbonate recycling.

**Figure 4.**
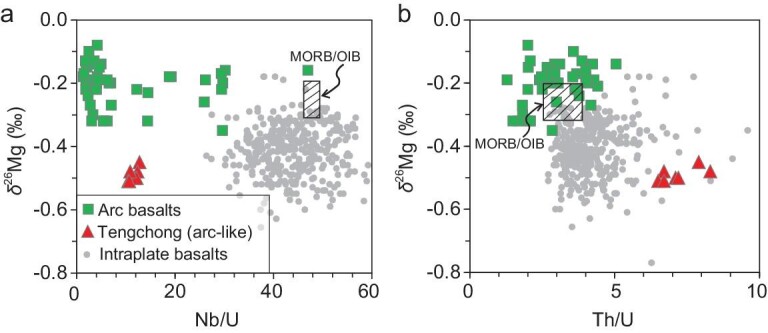
Plotting δ^26^Mg against (a) Nb/U and (b) Th/U for arc basalts, arc-like Tengchong basalts and intraplate basalts from Eastern China. The mantle source of arc basalts has been metasomatized by the aqueous fluids dehydrated from subducting slabs. The aqueous fluids have low Th/U, and low Nb/U because of rutile residual in the source during dehydration. The arc-like Tengchong basalts are derived from a back arc or continental margin mantle source modified by supercritical fluid with high Th/U but low Nb/U. The supercritical fluid can dissolve Mg-rich carbonate minerals and thus have high Mg and low δ^26^Mg features. The intraplate basalts from Eastern China have higher Nb/U and relatively high Th/U. They are from a mantle source that has been modified by melts of carbonates or carbonated eclogites from the subducting slab. Data for arc basalts are from Refs [1,64]; data for Tengchong basalts are from Ref. [65]; data for intraplate basalts from Eastern China are from Refs [1,55–59].

From trench to island arc depth (70–120 km), the fluids dehydrated from metasediments, metabasalts or metaperidotite are mainly aqueous fluids containing only a small amount of Mg compared to their sources. Thus, the loss of Mg by metamorphic dehydration does not cause any Mg isotopic changes in the metamorphic products. The aqueous fluids selectively dissolve calcite while leaving Mg-rich carbonate minerals in the subducting slab. Fluid infiltration also facilitates decarbonation and isotopic exchange between subducting silicate and carbonate at forearc and island arc depths. At this stage, most calcites in subducting slabs are either decarbonated or dissolved in aqueous fluids, releasing CO_2_ into arc volcanism, and some are transferred to Mg-rich carbonate minerals due to Ca-Mg exchange between silicate and carbonate. The latter can be delivered to the deep mantle by subducting slabs. The subducting silicates, when interacting with the carbonates (for example, carbonated eclogites), can obtain light Mg isotopic signatures. Although the fluid may evolve to be highly enriched in light Mg isotopes because of carbonate dissolution, its impact on the Mg isotopic system of the mantle wedge source of arc basalts is limited due to the remarkably lower Mg concentration compared to the peridotitic mantle. This can explain why most arc basalts with a source that has been modified by infiltration of such CO_2_-rich fluids do not usually display light Mg isotopic signatures (Fig. 4). The involvement of subducting sediments or sediment-derived melts in the mantle source gives the arc basalts mantle-like or slightly heavier Mg isotopic compositions [1,64].

With increasing temperature and pressure to the back arc or active continental margin system (>150 km to <410 km), supercritical fluid appears. In contrast to the aqueous fluid, the supercritical fluid has higher solubility of trace elements and carbonate minerals. In particular, the D_Th_^liquid/solid^ is >D_U_^liquid/solid^ in supercritical fluids but is <D_U_^liquid/solid^ in aqueous fluids [42]. Therefore, the supercritical fluid has higher Th/U than the aqueous fluid. In addition, a number of carbonate mineral inclusions, including calcite, dolomite and magnesite observed in metamorphic zircons precipitated from supercritical fluids in the Maowu massif of the Dabie orogen, suggest that supercritical fluid at mantle depth >150 km can dissolve more Mg-rich carbonate minerals but not rutile in eclogites [25]. The mantle wedge metasomatized by the supercritical fluids has δ^26^Mg down to −0.99‰ and Th/U ratio up to 23 [25]. The arc-like basaltic rocks generated from such mantle source (e.g. Tengchong basalt) [65] can be distinguished from typical arc basalts in terms of lower δ^26^Mg and higher Th/U (Fig. 4b). Both have low Nb/U ratios because of rutile residual in the subducted eclogites (Fig. 4b).

When the silicate-carbonate package is subducted to the mantle transition zone (410–660 km), both Mg-rich carbonates and carbonated eclogites can melt. Recycling and involvement of these components in the mantle source can account for the light Mg isotopic signatures observed in ocean island basalt (OIB)-like intraplate basalts. They are distinguishable from arc or arc-like basalts by their high Nb/U ratio and variably low δ^26^Mg values (Fig. 4a). However, it is still a puzzle whether the light Mg isotopic signatures result from recycling of carbonates or carbonated eclogites. Previous studies revealed that the low-δ^26^Mg basalts from New Zealand, Eastern China, Hainan Island, Vietnam and Pitcairn Island are related to the recycling of carbonated eclogites [1,55,58,66–68]. Wang *et al*. [66] first proposed that carbonated eclogite-derived melts are involved in the genesis of low-δ^26^Mg Antipodes Volcano basalts from New Zealand, based on the negative correlation between δ^26^Mg and Gd/Yb ratios. Li *et al*. [1] concluded that there are two low-δ^26^Mg components in the mantle of Eastern China and Hainan Island. The mantle of Eastern China is characterized by low Fe/Mn and high CaO/Al_2_O_3_ ratios that are consistent with carbonated peridotite-derived melts; the mantle of Hainan Island features high Fe/Mn and low CaO/Al_2_O_3_ ratios, which is indicative of recycled eclogite-derived melts. Future work coupling Mg isotopes to other major/trace element proxies is needed to further constrain how the low-δ^26^Mg signature is related to the deep carbon cycle. As a consequence, using the Mg isotopic system to quantify the proportion of recycled carbonate component in the mantle source is still in the early stages.

Other stable isotopic systems, such as Ca and Zn isotopes, have been increasingly applied to trace deep carbonate recycling [11] and shed more light onto the nature and fate of deep recycled carbon. However, the geochemical behavior of the silicate-carbonate system during crustal subduction remains poorly known. Before the stable isotopic systems of divalent metals in carbonates are put together to provide better constraints on deep carbonate cycling, the behavior of metal stable isotopes during subduction needs to be evaluated.
